# Hippocampal subfield segmentation in temporal lobe epilepsy: Relation to outcomes

**DOI:** 10.1111/ane.12926

**Published:** 2018-03-23

**Authors:** B. A. K. Kreilkamp, B. Weber, S. B. Elkommos, M. P. Richardson, S. S. Keller

**Affiliations:** ^1^ Department of Molecular and Clinical Pharmacology Institute of Translational Medicine University of Liverpool Liverpool UK; ^2^ Department of Neuroradiology The Walton Centre NHS Foundation Trust Liverpool UK; ^3^ Department of Epileptology University of Bonn Bonn Germany; ^4^ Center for Economics and Neuroscience University of Bonn Bonn Germany; ^5^ Department of NeuroCognition/Imaging Life& Brain Research Center Bonn Germany; ^6^ Department of Molecular and Clinical Sciences St George's, University of London London UK; ^7^ Department of Basic and Clinical Neuroscience Institute of Psychiatry, Psychology & Neuroscience King's College London London UK; ^8^ Engineering and Physical Sciences Research Council Centre for Predictive Modelling in Healthcare University of Exeter Exeter UK

**Keywords:** automated, hippocampal sclerosis, hippocampal subfield mapping, multisequence, surgical outcomes, temporal lobe epilepsy

## Abstract

**Objective:**

To investigate the clinical and surgical outcome correlates of preoperative hippocampal subfield volumes in patients with refractory temporal lobe epilepsy (TLE) using a new magnetic resonance imaging (MRI) multisequence segmentation technique.

**Methods:**

We recruited 106 patients with TLE and hippocampal sclerosis (HS) who underwent conventional T1‐weighted and T2 short TI inversion recovery MRI. An automated hippocampal segmentation algorithm was used to identify twelve subfields in each hippocampus. A total of 76 patients underwent amygdalohippocampectomy and postoperative seizure outcome assessment using the standardized ILAE classification. Semiquantitative hippocampal internal architecture (HIA) ratings were correlated with hippocampal subfield volumes.

**Results:**

Patients with left TLE had smaller volumes of the contralateral presubiculum and hippocampus‐amygdala transition area compared to those with right TLE. Patients with right TLE had reduced contralateral hippocampal tail volumes and improved outcomes. In all patients, there were no significant relationships between hippocampal subfield volumes and clinical variables such as duration and age at onset of epilepsy. There were no significant differences in any hippocampal subfield volumes between patients who were rendered seizure free and those with persistent postoperative seizure symptoms. Ipsilateral but not contralateral HIA ratings were significantly correlated with gross hippocampal and subfield volumes.

**Conclusions:**

Our results suggest that ipsilateral hippocampal subfield volumes are not related to the chronicity/severity of TLE. We did not find any hippocampal subfield volume or HIA rating differences in patients with optimal and unfavorable outcomes. In patients with TLE and HS, sophisticated analysis of hippocampal architecture on MRI may have limited value for prediction of postoperative outcome.

## 1 INTRODUCTION

Epilepsy is the most common serious neurological disorder. Refractory temporal lobe epilepsy (TLE) with hippocampal sclerosis (HS) is the most common medically intractable epilepsy condition.^1^ Temporal lobe surgery may render between 38 and 60% patients seizure free, depending on the time to postoperative follow‐up and definition of seizure freedom.^2^, ^3^, ^4^, ^5^ Quantitative magnetic resonance imaging (MRI) techniques provide sensitive surrogate markers of HS.^6^ Global hippocampal atrophy is most frequently quantified on T1‐weighted (T1w) MRI in patients with TLE. In patients with TLE and HS, hippocampal atrophy has been correlated with various clinical features of the disorder, including age of onset of intractable seizures, duration of epilepsy, and postoperative seizure outcome.^7^, ^8^, ^9^ However, other studies have failed to report these associations.^10^, ^11^ A potential reason for these discrepancies could be the fact that hippocampal volume alone is not a reliable predictor of post‐surgical outcome^11^, ^12^ or even of the presence^6^ or absence^13^ of HS. Indeed, hippocampal volume asymmetry has also been demonstrated in age‐matched healthy controls regardless of image presentation during manual measurements.^14^ Hippocampal internal architecture (HIA) and variation in hippocampal signal intensity should also be considered alongside volume in context of neuroradiological evaluation.^6^, ^8^, ^13^ Signal intensity assessment and semiquantitative HIA ratings are made based on high‐resolution coronal MR images, which provide high signal‐ and contrast‐to‐noise ratios. HIA ratings can indicate severity and type of HS and may reveal correlations with various clinical features of the disorder.^15^, ^16^ Consequently, identifying relationships between clinical features and quantitative characteristics of the hippocampus in TLE is important as they may offer insights into the pathophysiology of the disorder, interindividual patient heterogeneity, and may provide the basis for imaging prognostic markers of treatment outcome.

The International League Against Epilepsy (ILAE) Commission on Diagnostic Methods have reported three principle patterns of HS based on histopathological analysis.^17^ The most common pattern of cell loss, ILAE HS type 1, is manifest as predominant loss of neurons and gliosis in CA1 and CA4 subfields.^17^, ^18^ ILAE HS type 2 and 3 are less common patterns of HS, manifest as pathological changes predominantly in CA1 or CA4, respectively.^17^, ^18^ Importantly, these patterns of HS appear to be related to various clinical aspects of TLE and may have significance for postoperative prognosis. ILAE HS type 1 is more frequently associated with a history of initial precipitating injuries in early childhood, an early seizure onset and improved seizure outcome after temporal lobe surgery.^17^, ^18^, ^19^, ^20^ ILAE HS type 2 and 3 appear to be associated with a later age of onset and a less favorable postoperative outcome,^17^, ^18^, ^19^, ^20^ although there are some inconsistencies in these relationships.^21^ Given the clinical relevance of regional hippocampal subfield pathology in TLE, it is important to develop and apply MRI approaches that permit assessment of hippocampal subfield structure and volume in this patient group, particularly if such non‐invasive imaging measures can be used to predict treatment outcome.

There have been significant advances in the development of MRI techniques for the segmentation and volume estimation of hippocampal subfields. Manual delineation techniques applied to high‐field (ie, ≥ 4 Tesla) MRI are the most reliable approaches to identify the approximate location of subfields in individual subjects.^22^, ^23^, ^24^ Automated hippocampal subfield approaches applied to high‐field MRI have also been described.^25^ However, applications of these approaches are constrained by the necessity of non‐clinical high‐field MRI scanners and the time‐inefficient manner of manual tracing. There have therefore been developments of automated hippocampal subfield techniques that can be applied to clinically acquired (ie, ≤ 3 Tesla) MRI data.^26^, ^27^ The approach described by Van Leemput et al. (2009) has proved to be particularly popular, given this method's release in context of the freely available Freesurfer software (http://freesurfer.net).^28^ We have previously applied this technique to investigate hippocampal subfield alterations in patients with TLE.^29^ However, there have been concerns raised with this approach, including reliance on low‐resolution T1w images and an imprecise parcellation scheme.^30^ Recently, a revised automated hippocampal subfield technique has been introduced that has improved anatomical delineation of the constituent parts of the hippocampus based on multisequence MRI, including standard resolution T1w images and high in‐plane resolution T2w images.^31^ In a large sample of patients with refractory TLE and HS who underwent conventional T1w and high‐resolution T2 short TI inversion recovery (T2STIR) MRI, we have applied this latest approach to investigate whether preoperative in vivo hippocampal subfield analysis had significance for the side of seizure onset, postoperative seizure control, semiquantitative HIA ratings, and other clinical features of TLE.

## 2 METHODS

### 2.1 Participants

We studied 106 patients with well‐characterized mesial TLE and radiological evidence of HS (mean age 40.3 years (SD 13.6); 59 female; 67 with left TLE, 39 with right TLE) who were being evaluated for suitability for neurosurgery at University Hospital Bonn, Germany. Each patient underwent a detailed presurgical program, including comprehensive seizure semiology assessment, MRI, neuropsychological assessment, interictal electroencephalography and if clinically necessary, additional invasive electrophysiological recordings, as reported recently.^10^, ^32^ All patients showed evidence of a unilateral temporal lobe seizure onset with concomitant ipsilateral HS. HS was identified by an expert neuroradiologist with considerable experience in lesion diagnosis in epilepsy, which was defined by hippocampal volume loss and internal structure disruption on T1w images, and/or hyperintensities on T2w and FLAIR images.^10^ There was no evidence of bilateral HS in any patient; all patients had seizures of presumed unilateral temporal lobe origin, and there was no evidence of a secondary extrahippocampal lesion that may have contributed to seizures.^32^ All patients underwent standardized amygdalohippocampectomy and routine diagnostic analysis of resected hippocampal specimens by an experienced neuropathologist.^10^ Histological assessment of resected hippocampal specimens revealed that 83% had HS ILAE type I and 17% had HS ILAE type II, no patient had HS ILAE type III.^33^ Age of patient, age at diagnosis of epilepsy, duration of epilepsy, history of childhood febrile convulsions, and incidence of secondary generalized tonic‐clonic seizures (SGTCS) were recorded for all patients. Patients who underwent temporal lobe surgery (standardized amygdalohippocampectomy) received postoperative follow‐up for a period of up to 2 years after surgery and outcome assessment using the ILAE outcome classification system.^34^

### 2.2 MRI acquisition

All patients underwent MRI at the Life & Brain Center in Bonn on a 3 Tesla scanner (Magnetom Trio, Siemens, Erlangen, Germany) using an 8‐channel head coil. For the purposes of this study, we acquired two MRI sequences, including a 3D T1w MPRAGE image (160 slices, TR = 1300 ms, TI = 650 ms, TE = 3.97 ms, resolution 1.0 × 1.0 × 1.0 mm, flip angle 10°) and a high in‐plane resolution T2STIR sequence in the coronal plane angulated perpendicular to the long axis of the hippocampus (40 slices, TR = 5600 ms, TI = 100 ms, TE = 18 ms, resolution .45 × .45 × 2.0 mm, flip angle 0°). For 50 patients undergoing resective surgery, T1w images were also acquired after surgery.

### 2.3 MRI analysis

**Figure 1:**
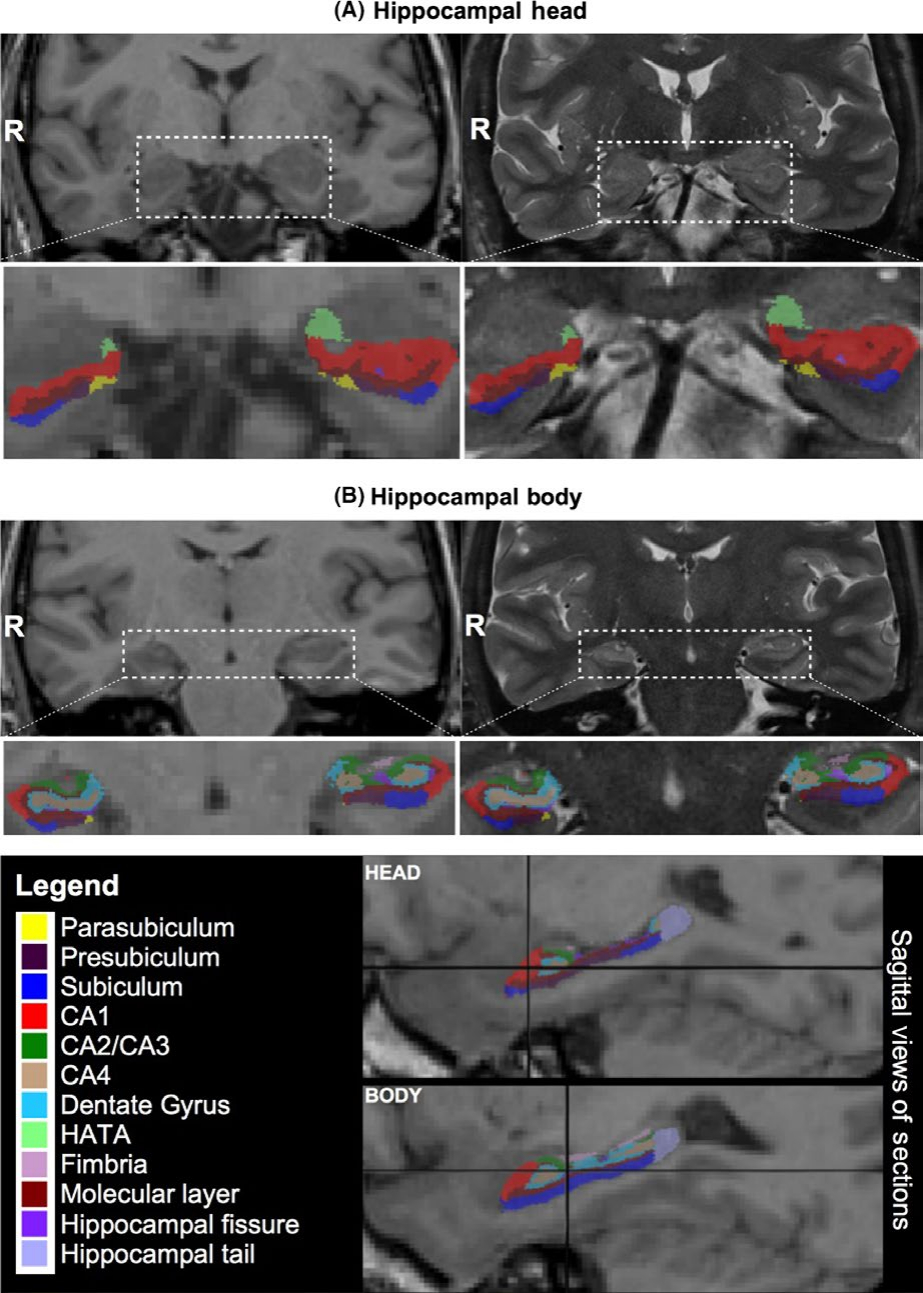
Anatomical locations of segmented subfields on T1w (left) and T2STIR images (right) in a patient with right TLE. The same anatomical slices are shown for both images in the hippocampal head (A) and hippocampal body (B). R  =  right

For each patient, we performed quantitative automated segmentation and cortical parcellation of T1w data using Freesurfer version 5.3.0.^28^ The standard Freesurfer “recon‐all” processing stream was used, which provides surfaces and morphometry data for each subject in addition to gray and white matter segmentations. Automatic labeling and volume estimation of hippocampal subfields were guided by the segmentation of the whole hippocampus (previous step) and performed using the adaptive segmentation technique described by Iglesias et al (2015) in context of the published Freesurfer software version 6 (https://surfer.nmr.mgh.harvard.edu/fswiki/HippocampalSubfields). Figure 1 shows the anatomical locations of the hippocampal subfields on T1w and T2STIR images in a patient with right TLE after the use of this software module. The protocol coregistered T1w and T2STIR data and used these images simultaneously to generate labels and volumes for the whole hippocampus and 12 hippocampal subfields:

1. Parasubiculum
2. Presubiculum
3. Subiculum
4. CA1
5. CA2/CA3
6. CA4
7. Granule cell layer of the dentate gyrus (GC‐DG)
8. Hippocampus‐amygdala transition area (HATA)
9. Fimbria
10. Molecular Layer
11. Hippocampal fissure
12. Hippocampal tail

Asymmetry indices for ICV‐corrected hippocampal volumes were computed using the previously published formula.^35^ Semiquantitative HIA ratings have been shown to be a significant predictor of the laterality of seizure onset in TLE^17^, ^33^ and were integrated into image analysis in order to determine if HIA correlates with gross hippocampal and subfield volumes as estimated by Freesurfer version 6. Each T2STIR image slice that depicted the hippocampus was graded with a score of “1” when no internal architecture was perceptible to “4” where excellent internal architecture differentiation could be appreciated.^33^ The rater (S.B.E.) was blinded to patient clinical information such as outcome and laterality, and the images were rated on consecutive coronal T2STIR sections in a rostral to caudal direction as described in our previous study.^33^ An analysis including resection volumes has been previously performed on this data by the authors and has been fully described.^10^

### 2.4 Statistical analysis

All statistical analyses were performed using MATLAB 2015b. Group comparison analyses were performed using the unpaired Mann‐Whitney U test (data non‐normally distributed, *P* < .05) and included analysis of effects of laterality of epilepsy and postoperative outcome on subfield volume. With respect to postoperative outcome, comparisons were made between patients who attained a postoperative outcome of ILAE 1 (complete seizure freedom) relative to ILAE 2 +  (persistent postoperative seizure‐related symptoms).^10^ Relationships between subfield volume/asymmetry indices and clinical data, including age of onset of epilepsy, epilepsy duration, seizure frequency, and estimated seizure burden, were investigated using Spearman correlation coefficients. Seizure burden was defined as equal to log_10_(*frequencyxduration*), with the logarithm being applied to accommodate patients with very high‐seizure frequency. Correlations were performed corrected for patient age. Relationships between categorical relationships, including postoperative outcome and sex, side of TLE, and history of childhood febrile/SGTC seizures, were investigated using chi‐squared tests of independence. Furthermore, we investigated relationships between HIA ratings and automatically extracted subfield volumes. Asymmetry indices, gross hippocampal, and subfield volumes were corrected for intracranial volume (ICV), and statistical tests were corrected for multiple comparisons using the false‐discovery rate (FDR) procedure.

## 3 RESULTS

The accuracy of the hippocampal subfield labels was visually checked for all patients. The subfields of one hippocampus in three patients could not be successfully generated. Therefore, analyses were restricted to the 103 with successful reconstructions.

### 3.1 Volumes and clinical correlations

**Figure 2:**
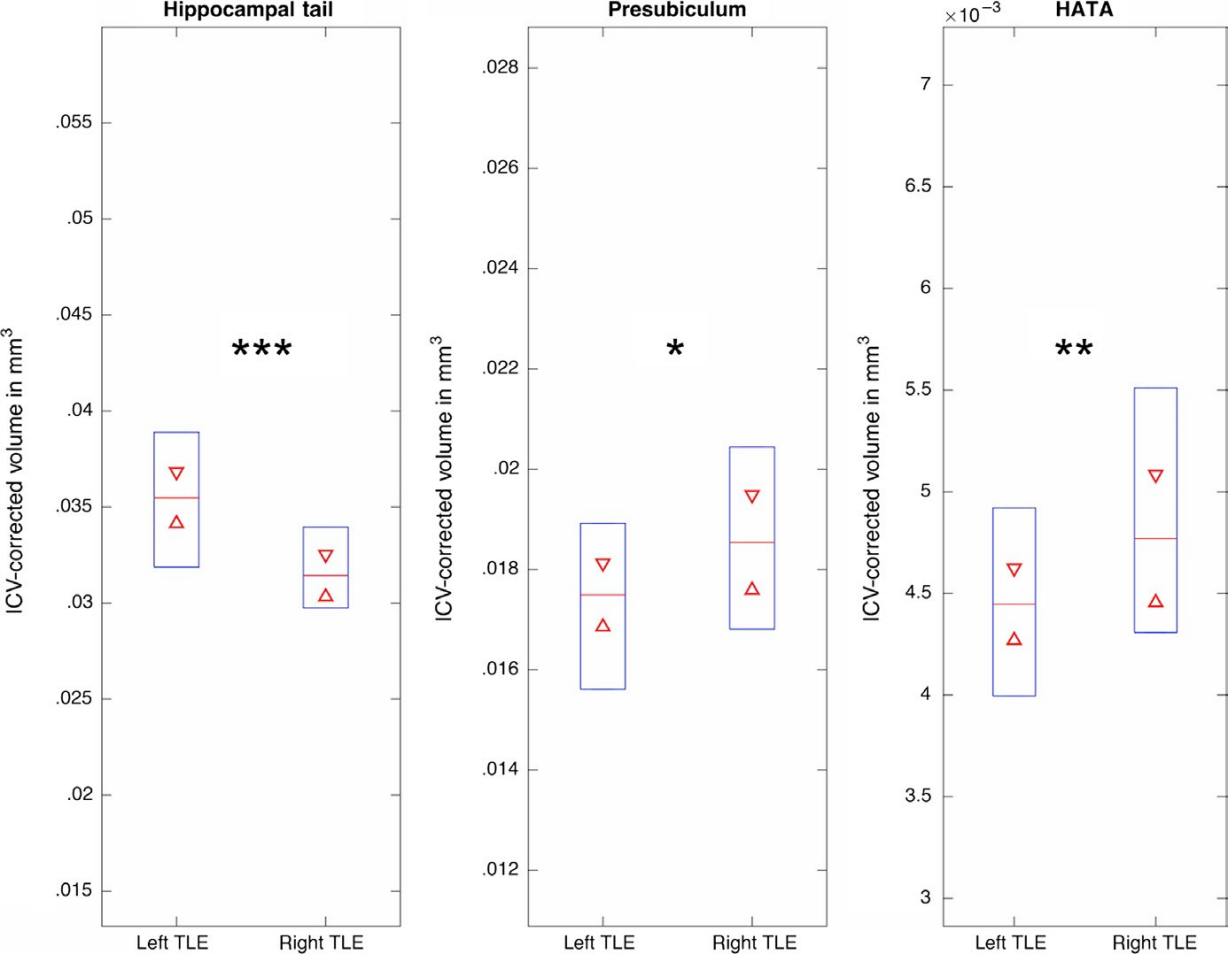
Decreased contralateral hippocampal volumes in patients with right TLE compared to patients with left TLE (Hippocampal Tail) and vice versa (Presubiculum/HATA). Blue boxplots indicate data distribution, with the median (red line) and 95% confidence intervals (red triangles). *P < .1; **P < .05; ***P < .001

Table 1 shows the comparison of ipsilateral and contralateral subfield volumes between patients with left and right TLE. There were no significant differences in subfield volumes of the ipsilateral hippocampus between patients with left and right TLE. Patients with right TLE had significantly reduced ICV‐corrected volumes of the contralateral hippocampal tail (Z = 3.3, *P*_(FDR‐corr.)_=.01), and patients with left TLE had significantly reduced volumes of the contralateral presubiculum (Z = −2.4, *P*_(FDR‐corr.)_ = .08) and HATA (Z = −2.66, *P*_(FDR‐corr.)_ = .05) relative to the corresponding patient group (Table 1; Figure 2). For patients as a whole group, and patients with left and right TLE separately, there were no significant relationships between age of onset, duration of epilepsy corrected for age, seizure frequency/burden, incidence of SGTCS/febrile convulsions, and hippocampal subfield volumes (*P*_(FDR‐corr)_ > .05). These clinical variables did not correlate with ICV‐corrected hippocampal asymmetry indices (*P*_(FDR‐corr)_ > .05).

**Table 1:** Comparison of subfield volumes corrected for ICV in patients with left/right TLE

Side	Region	TLE	Mean	SD	Z	*P*‐value (FDR‐corr.)
Ipsilateral	Hippocampal Tail	Left	.025	.007	.15	.96
		Right	.025	.005		
	Subiculum	Left	.022	.005	1.89	.37
		Right	.020	.003		
	CA1	Left	.032	.009	−.72	.76
		Right	.032	.007		
	Hippocampal Fissure	Left	.008	.002	1.3	.53
		Right	.008	.002		
	Presubiculum	Left	.015	.004	2.6	.11
		Right	.014	.003		
	Parasubiculum	Left	.003	.001	1.3	.53
		Right	.003	.001		
	Molecular Layer HP	Left	.029	.006	.32	.96
		Right	.028	.005		
	GC‐ML‐DG	Left	.015	.004	.21	.96
		Right	.015	.003		
	CA2/3	Left	.010	.003	−.85	.74
		Right	.010	.003		
	CA4	Left	.013	.003	.05	.96
		Right	.012	.003		
	Fimbria	Left	.004	.001	1.48	.53
		Right	.004	.001		
	HATA	Left	.004	.001	1.16	.53
		Right	.004	.001		
	Whole Hippocampus	Left	.170	.040	.46	.93
		Right	.166	.030		
Contralateral	Hippocampal Tail	Left	.036	.006	3.3	.01
		Right	.032	.005		
	Subiculum	Left	.028	.005	−1.02	.52
		Right	.028	.004		
	CA1	Left	.045	.008	−1.7	.32
		Right	.043	.006		
	Hippocampal Fissure	Left	.088	.002	.74	.55
		Right	.084	.002		
	Presubiculum	Left	.018	.003	−2.4	.08
		Right	.019	.003		
	Parasubiculum	Left	.004	.001	−1.23	.52
		Right	.004	.001		
	Molecular Layer HP	Left	.038	.007	1.14	.52
		Right	.036	.005		
	GC‐ML‐DG	Left	.021	.004	.43	.67
		Right	.021	.003		
	CA2/3	Left	.015	.003	.58	.61
		Right	.015	.003		
	CA4	Left	.018	.003	.82	.54
		Right	.017	.002		
	Fimbria	Left	.005	.001	−.88	.54
		Right	.005	.002		
	HATA	Left	.0045	.001	−2.66	.05
		Right	.005	.001		
	Whole Hippocampus	Left	.230	.036	.99	.52
		Right	.225	.028		

### 3.2 Outcome

Of the 103 with successful reconstructions, 76 patients had received amygdalohippocampectomy and postoperative outcome assessment. Of these patients, 41 (54%) patients were seizure free postoperatively (ILAE 1), and 35 (46%) had persistent seizure symptoms (ILAE 2 + ) (minimum 12‐month follow‐up, mean 23 months). A breakdown of clinical variables according to outcome is provided in Table 2. An increased number of males was rendered seizure free compared with females (χ^2^ = 4.5, *P* < .05), and right‐sided patients were less likely to experience postoperative seizures (χ^2^ = 3.7 *P* = .05). There were no significant differences between outcome groups in incidence of febrile/SGTC seizures, age, age at onset of epilepsy, duration of epilepsy, seizure frequency, or seizure burden. When all patients were considered together, there were no significant differences in the volume of ipsilateral or contralateral hippocampal subfields between those with postoperative seizure freedom and those with persistent seizure symptoms (*P*_(FDR‐corr)_ > .05; Table 3). There was also no difference between outcome groups with respect to ICV‐corrected hippocampal asymmetry indices (*P*_(FDR‐corr)_ > .05). No significant differences were observed between outcome groups when patients with left or right TLE were considered separately. There was also no correlation between the extent of resection and outcome.^10^

**Table 2:** Clinical variables according to surgery outcome

	ILAE 1	ILAE 2+	Statistics
N	41 (54%)	35 (46%)	‐
Outcomes	1 = 41	2 = 7 3 = 14 4 = 11 5 = 3 6 = 0	‐
Left/Right TLE	23/18	27/8	χ^2^ =3.7, *P* = .05
Female/Male	17/24	23/12	χ^2^ =4.5, *P* = .04
Febrile Seizures, no/yes	28/13	21/14	χ^2^ =.6, *P* = .45
SGTCS, no/yes	25/16	21/14	χ^2^ =.01, *P* = .93
Age	38.3 (12.4)	39.1 (14.2)	Z=−.08, *P* = .94
Onset	15.9 (11.94)	14.9 (11.5)	Z = .27, *P* = .79
Duration Corrected for Age	.58 (.29)	.59 (.3)	Z=−.06, *P* = .95
Seizure Frequency	8.8 (16.5)	8.4 (15.6)	Z=−.75, *P* = .45
Seizure Burden	1.87 (.52)	1.89 (.52)	Z=−.14, *P* = .89

TLE, temporal lobe epilepsy; SGTCS, secondary generalized tonic‐clonic seizures.

**Table 3:** Comparison of subfield volumes corrected for ICV in patients with outcomes ILAE 1 vs ILAE 2+

Side	Region	Outcome	Mean	SD	Z	*P*‐value (FDR‐corr.)
Ipsilateral	Hippocampal Tail	ILAE 1	.026	.006	−.570	.690
		ILAE 2+	.026	.007		
	Subiculum	ILAE 1	.022	.005	−.830	.690
		ILAE 2+	.022	.005		
	CA1	ILAE 1	.033	.009	−.780	.690
		ILAE 2+	.033	.008		
	Hippocampal Fissure	ILAE 1	.008	.002	.323	.750
		ILAE 2+	.008	.002		
	Presubiculum	ILAE 1	.015	.004	−1.271	.690
		ILAE 2+	.015	.003		
	Parasubiculum	ILAE 1	.003	.001	−2.011	.580
		ILAE 2+	.004	.001		
	Molecular Layer HP	ILAE 1	.029	.006	−.552	.690
		ILAE 2+	.029	.006		
	GC‐ML‐DG	ILAE 1	.015	.004	−.667	.690
		ILAE 2+	.015	.004		
	CA2/3	ILAE 1	.011	.003	−.323	.750
		ILAE 2+	.011	.003		
	CA4	ILAE 1	.013	.004	−.552	.690
		ILAE 2+	.013	.003		
	Fimbria	ILAE 1	.004	.001	−.761	.690
		ILAE 2+	.004	.001		
	HATA	ILAE 1	.004	.001	−.866	.690
		ILAE 2+	.004	.001		
	Whole Hippocampus	ILAE 1	.174	.042	−.865	.690
		ILAE 2+	.177	.039		
Contralateral	Hippocampal Tail	ILAE 1	.034	.005	−.020	.980
		ILAE 2+	.035	.008		
	Subiculum	ILAE 1	.028	.004	.560	.980
		ILAE 2+	.028	.005		
	CA1	ILAE 1	.044	.007	−.410	.980
		ILAE 2+	.045	.009		
	Hippocampal Fissure	ILAE 1	.087	.002	−.230	.980
		ILAE 2+	.089	.002		
	Presubiculum	ILAE 1	.018	.002	.150	.980
		ILAE 2+	.018	.004		
	Parasubiculum	ILAE 1	.004	.001	−1.060	.980
		ILAE 2+	.004	.001		
	Molecular layer HP	ILAE 1	.037	.006	−.220	.980
		ILAE 2+	.038	.007		
	GC‐ML‐DG	ILAE 1	.021	.004	−.896	.980
		ILAE 2+	.021	.004		
	CA2/3	ILAE 1	.015	.003	−.125	.980
		ILAE 2+	.015	.003		
	CA4	ILAE 1	.017	.003	−.750	.980
		ILAE 2+	.018	.003		
	Fimbria	ILAE 1	.005	.001	.042	.980
		ILAE 2+	.005	.001		
	HATA	ILAE 1	.005	.001	−.021	.980
		ILAE 2+	.005	.001		
	Whole Hippocampus	ILAE 1	.229	.032	−.240	.980
		ILAE 2+	.231	.039		

### 3.3 Subfield volumes and HIA ratings

**Figure 3:**
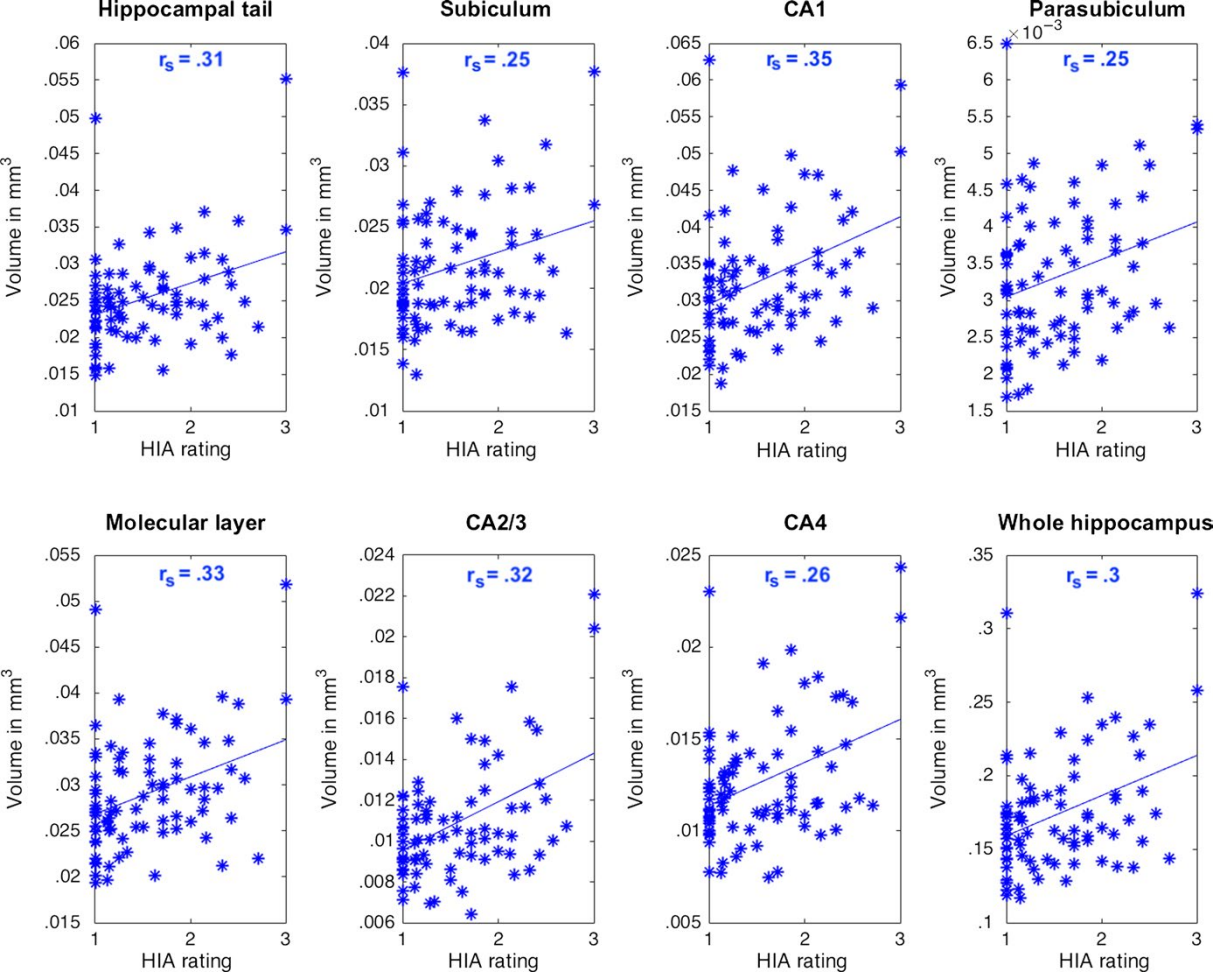
Significant correlations of ipsilateral hippocampal internal architecture (HIA) ratings and ipsilateral subfield volumes extracted via Freesurfer. Linear least‐square lines were fitted to the data

Significant correlations were observed between semiquantitative ipsilateral HIA ratings and ipsilateral hippocampal tail (r_s_ = .31; .35; *P*_(FDR‐corr.)_ < .05), parasubiculum (r_s_ = .25; *P*_(FDR‐corr.)_ < .05), molecular layer (r_s_ = .33; *P*_(FDR‐corr.)_ < .05), CA2/3 (r_s_ = .32; *P*_(FDR‐corr.)_ < .05), CA4 (r_s_ = .26; *P*_(FDR‐corr.)_ < .05), and whole hippocampal volume (r_s_ = .3; *P*_(FDR‐corr.)_ < .05). These relationships are shown in Figure 3. There were no correlations between contralateral HIA ratings and contralateral hippocampal or subfield volumes (*P*_(FDR‐corr)_ > .05).

## 4 DISCUSSION

The present study is the first to use a novel multicontrast approach to improve automated hippocampal subfield segmentation in TLE and to relate these measures to HIA ratings and clinical features. In this study, we have reported four primary findings. Firstly, patients with left TLE had significantly decreased volume of the contralateral presubiculum and HATA regions relative to patients with right TLE. Conversely, patients with right TLE had significantly smaller contralateral hippocampal tail volumes relative to patients with left TLE. Secondly, ipsilateral and contralateral hippocampal subfield volumes did not correlate with duration of epilepsy corrected for age, age of onset of epilepsy, epilepsy burden, a history of febrile seizures, or prevalence of SGTCS. Thirdly, the volume of ipsilateral or contralateral hippocampal subfields was not associated with postoperative seizure outcome. Finally, HIA ratings were significantly related to several subfield volumes of the pathological hippocampus. We discuss the biological and clinical significance of these findings before highlighting the strengths and limitations of this work.

### 4.1 Biological and clinical implications

Although left and right TLE do not differ in the extent of atrophy of the epileptogenic hippocampal subfields, patients with left TLE had significantly reduced volumes of the contralateral parasubiculum and HATA regions relative to patients with right TLE. This is a new finding and suggests that left TLE may be associated with a bihemispheric hippocampal subfield alterations in these particular regions compared with right TLE, who showed evidence of reduced volumes in the contralateral hippocampal tail relative to patients with left TLE. There is an inconsistent literature on the effects of TLE laterality on the distribution of brain damage, with some indicating increasingly bilateral changes in left TLE,^36^, ^37^ in right TLE^35^, ^38^ and some studies suggesting equivalence.^39^ Just one of these studies^39^ has entered the hippocampal asymmetry (right>left) found in healthy controls^14^ as a confounding factor during statistical analysis. It is possible that natural cerebral asymmetry of this structure may account for some differences found in patients.^14^ Unfortunately, as T2STIR images were not available for our healthy controls, we are unable to resolve this, so that this would be a worthwhile addition to future studies.

Whether recurrent seizures cause progressive brain damage is a contentious issue. In the absence of longitudinal data, cross‐sectional studies have correlated brain compartment volume with duration of epilepsy as a surrogate marker of progressive damage due to seizure chronicity. There is inconsistency in the literature with respect to relationships between hippocampal and extrahippocampal volume loss and duration of TLE.^7^, ^10^, ^11^, ^36^ Given that duration of epilepsy and chronological age are related, it is important to correct clinical correlations for patient age to determine whether brain atrophy is driven by epilepsy‐related factors or normal age‐related maturation. In the present study, we report that subfields of the epileptogenic and contralateral hippocampus are not correlated with clinical variables. These results suggest that hippocampal subfields are not susceptible to damage due to the general chronicity of the disorder or age of onset. This is consistent with previous work that indicates that HS may be a direct and immediate consequence of an initial precipitating injury (eg, febrile seizures/infection/genetic defects); hippocampal subfield volume loss may not be primarily influenced by the chronicity/severity of the disorder as the majority of neuron loss occurs during epileptogenesis before onset of seizures.^40^, ^41^ It is worth noting that one study reported a relationship between a longer duration of epilepsy corrected for age and volumes of left CA1, presubiculum, and subiculum in left TLE and right CA1 in right TLE.^42^ There is evidence from cross‐sectional studies that progressive hippocampal atrophy may occur in patients with TLE; however, longitudinal multicohort studies are needed to differentiate between normal aging and disease progression.^43^

With respect to postoperative outcome, harmonizing results from histopathological studies of hippocampal subfield neuronal loss and imaging studies of hippocampal subfield volume loss is difficult because of the inherent differences in the resolution of tissue characteristics. Postoperative outcome has been shown to be superior in patients with TLE who, after resection, were retrospectively shown to have classical patterns of HS (ie, preservation of CA2 neurons) or total HS (ie, neuronal loss throughout the CA), whereas patients with circumscribed neuronal loss of CA1 or CA4 tend to have poorer outcomes.^17^, ^18^, ^19^, ^20^ Mathern et al (1996) had previously reported that patients with initial precipitating injuries were more likely to benefit from surgery and had neuronal cell loss in CA1 and presubiculum regions. We were unable to identify volume differences in preoperative hippocampal subfields between 41 patients with postoperative seizure freedom and 35 patients with persistent seizure symptoms. This is likely due to the fact that there is histopathological variability in HS across patients that is not identifiable on MRI. To varying degrees, patients with left and right TLE both showed contralateral volume reduction, and this may be interpreted to indicate increased bilateral mesial temporal damage, potentially reflecting a bihemispheric seizure disorder, which would be less amenable to surgical intervention. Specifically, contralateral hippocampal subfield volumetric changes confined to certain regions in patients with right or left TLE may have differential impact on surgical outcomes.

### 4.2 Strengths and limitations

The higher‐resolution multicontrast approach used in the present study clearly provided an improved segmentation of hippocampal subfields compared with an automated approach based solely on T1w images.^29^ There are, however, important considerations that should be made. Firstly, it should be emphasized that it is currently impossible to obtain an estimate of neuronal density from MRI determined hippocampal subfields, and the goal of in vivo imaging methods is to obtain an estimate volume of the approximate location of subfields. This likely explains the discrepancy between histopathology‐outcome correlations and imaging‐outcome correlations in patients with TLE. The combination of standard T1w images with a higher‐resolution T2 sequence, as applied in the present study, improves the delineation of the approximate location of the subfields. A meta‐analysis of studies reporting hippocampal subfield neuronal loss revealed statistically significant neuronal loss in all CA regions in patients with HS relative to control specimens, and CA1 was preferentially affected.^44^ It is an important point to consider hippocampal subfields or regions separately when analyzing hippocampal volumetrics based on presurgical MRI in relation to outcome. Unfortunately, due to the lack of healthy control data, we were unable to assess relationships between outcome and different types of HS as assessed by automated hippocampal subfield mapping. We did not have healthy control data in the present study because the high‐resolution T2STIR sequence was acquired only for patients being considered for surgery. Even though all of our patients were deemed to have unilateral HS, quantitative analysis of subfields did reveal some contralateral changes in the group of patients with left/right TLE.

Furthermore, hippocampal subfield mapping might be influenced by differences in image quality and motion across subjects.^31^ Some boundaries between structures might not be easily delineated given that some interfaces could not be detected in the training data.^45^ Patients received individual epilepsy surgery as clinically indicated, for example, with a trans‐sylvian (~50%) or subtemporal (~50%) access to the pathologic hippocampus, which was then removed in sections (trans‐sylvian) or in its entirety (subtemporal). Within this archival study, detailed microscopic information of intact hippocampal subfields from surgical specimens was not available for comparison with the automatically delineated hippocampal subfields based on MRI. However, this study is useful as it shows that ipsilateral semiquantitative HIA ratings were significantly related to automatically extracted ipsilateral hippocampal subfield volumes. This is an important finding and may indicate that automatic subfield mapping may be used for the detection of HS. In the absence of a gold‐standard procedure for in vivo mapping of hippocampal subfield volumes on 3T, the automated technique based on 7T ex vivo data may provide diagnostic utility in all patients with refractory epilepsy for the identification of HS, especially when individual patient volumes are compared with those of controls. An automated technique for hippocampal volume extraction that is sensitive to different patterns of HS could provide supplementary diagnostic information in a reproducible and time‐efficient way prior to surgery, especially when MRIs are otherwise unrevealing.

## 5 CONCLUSION

Different subtypes of HS have been shown to relate to postoperative outcome in patients with refractory TLE based on analysis of surgically resected specimens. For these findings to have prospective predictive utility, they need to be translated to imaging approaches that can assess hippocampal subfields ahead of surgery. The present study has applied a novel automated multisequence hippocampal subfield segmentation technique to a large group of patients with refractory TLE. This approach and semiquantitative HIA ratings did not reveal a clear link between hippocampal structure and postoperative outcome in patients with TLE and HS. Therefore, hippocampal features extracted from MRI are perhaps unlikely to stratify patients according to outcome, at least in this patient group. However, automated morphometry of hippocampal subfields may provide useful supplementary diagnostic utility in patients with no obvious evidence of HS.

## CONFLICT OF INTEREST

The authors declare that there are no conflict of interests in this study.
